# Exploratory Evaluation of Self‐Reported Periodontitis Among Adult Population From Comitán Chiapas, Mexico 

**DOI:** 10.1002/cre2.70274

**Published:** 2026-01-09

**Authors:** José A Falcón‐Flores, María E Jiménez‐Corona, Ileana G Rangel‐Nieto, Marisela Vazquez‐Duran, Aida Jiménez‐Corona

**Affiliations:** ^1^ Department of Ocular Epidemiology and Visual Health Institute of Ophthalmology Conde de Valenciana IAP Mexico City Mexico; ^2^ Department of Epidemiology Ignacio Chavez National Institute of Cardiology Mexico City Mexico; ^3^ Faculty of Medicine National Autonomous University of Mexico Mexico City Mexico; ^4^ General Directorate of Epidemiology Mexico City Mexico

**Keywords:** periodontitis, reliability, self‐report, validity

## Abstract

**Objective:**

We assessed the reliability and validity of a self‐report instrument to ascertain periodontitis risk.

**Materials and Methods:**

A cross‐sectional population‐based study was conducted in 2013 involving 454 adult people. The instrument included items on periodontal status such as self‐perception of gum health, bleeding and gingival infection, halitosis, tooth mobility, and tooth loss. The periodontal clinical condition was assessed using the Periodontal Screening and Recording Index. Construct validity was evaluated by exploratory factor analysis (EFA). Sensitivity, specificity, and area under the receiver operating characteristic curve (AUC‐ROC) were calculated as well. Reliability was assessed using Cronbach's alpha.

**Results:**

The prevalence of clinically evaluated moderate (MP) and severe (SP) periodontitis was 49.3% and 28.9%, respectively. Two factors for both types of periodontitis were identified using EFA. The combination of self‐report items and risk factors for periodontitis showed an AUC‐ROC of 0.660 (sensitivity 97.3%, specificity 3%) for MP and 0.804 (sensitivity 96.9%, specificity 28.3%) for SP.

**Conclusions:**

EFA showed two factors that accounted for the baseline and outcome stage of periodontitis and better predicted the risk of SP. This instrument can be an alternative for monitoring this disease at the population level.

## 1 Introduction

Periodontitis is an oral disease that damages the tissues surrounding and supporting the teeth; its final outcome is tooth loss. Surveillance of this condition is important to identify trends over time and plan strategies for its prevention and control (Beltrán‐Aguilar et al. 2012). Currently, the gold standard for periodontitis surveillance requires standardized clinical measurements of clinical attachment loss and probing depth (Holtfreter et al. 2015). Yet, in epidemiological studies it is not always possible to perform a complete clinical evaluation of periodontal status because of difficulties such as lack of highly trained personnel (Holtfreter et al. 2015).

The use of instruments for self‐report of periodontitis signs and symptoms has been proposed as a tool to carry out epidemiological surveillance, as it requires less time and resources. Some countries, such as Brazil, Nigeria, and India (Balappanavar et al. 2011; Cyrino et al. 2011; Akaji et al. 2010) have developed such instruments; however, they present a wide variability regarding the number of questions and the definitions of periodontitis used as a gold standard.

The National Center for Disease Control and Prevention (CDC) and the American Academy of Periodontology developed eight questions to predict the prevalence of periodontitis. Their efficacy was most useful for predicting severe periodontitis according to area under the receiver operating characteristic curve (AUC‐ROC) = 0.90, with a specificity value higher than the sensitivity (98% and 64%, respectively). Six‐site periodontal examination of all teeth was used as the gold standard (Eke and Dye 2009). Different studies have evaluated the efficacy of these questions in other languages (Carra et al. 2018; Iwasaki et al. 2021; Kapellas et al. 2021; Deng et al. 2021), including Spanish (Montero et al. 2020). They have proved useful, according to the defined cut‐off points (76.4% sensitivity and 63.5% specificity) for estimation of the prevalence of severe periodontitis (Montero et al. 2020). The questions, however, need to be evaluated in different populations, considering social characteristics such as education level, area of residence, and access to oral care.

Therefore, our goal was to assess the validity and reliability of a self‐report questionnaire to identify the risk of moderate periodontitis (MP) and severe periodontitis (SP) among the adult population in the Municipality of Comitán de Domínguez, Chiapas, Mexico.

## 2 Methods

### 2.1 Study Population

The Comitán study is a population‐based cross‐sectional study whose main goal was to assess the prevalence of type 2 diabetes among the population of the Municipality of Comitán de Domínguez in Chiapas, between 2010 and 2012. A detailed description of the study has been published previously (Jimenez‐Corona et al. 2019). Briefly, a census was conducted in three urban and five rural settings selected at random and for convenience, respectively. For the present analysis, three rural and two urban areas were selected for convenience. A total of 1940 people were examined. People with type 1 diabetes, pregnant women, and people with total edentulism were excluded. In all, 454 men and women aged ≥ 20 years at the time of the interview were randomly chosen and clinically assessed from July to October 2013. The evaluation included questionnaires on sociodemographic data and self‐reported periodontal status. A clinical oral examination was performed where the number of natural teeth (excluding third molars), oral hygiene, and periodontitis were assessed. The Research, Ethics, and Biosafety Committees of the National Institute of Public Health approved the study protocol. All participants signed an informed consent form.

### 2.2 Sociodemographic Variables

The variables of study were age, sex, area of residence, schooling (equal to or greater than primary/none), self‐reported indigenous origin, and occupation (housekeeper, farmer, merchant, or other). Rural settlements were defined according to criteria of Mexico's National Institute of Statistics and Geography (INEGI) as those with 2499 inhabitants or less. Diabetes *mellitus* was defined as a fasting serum glucose concentration ≥ 126 mg/dl or glucose at 2 h after load of 75 g of anhydrous glucose ≥ 200 mg/dl or with a previous medical diagnosis. Tobacco use was defined by self‐report, and participants were categorized as nonsmoker, ex‐smoker, and current smoker. Lastly, the use of dental services in the past 12 months and brushing frequency per day (one/two/three or more times) were evaluated.

### 2.3 Periodontal Status by Self‐Report

The instrument for periodontal assessment was designed using previously published self‐report periodontal status items, with apparent validity associated with signs and symptoms of periodontitis such as bleeding and gingival infection, self‐perception of gum health, halitosis, tooth mobility, and tooth loss (Eke and Dye 2009; Blicher et al. 2005). The wording, grammar, and definitions of the items selected for this study were evaluated considering the context in which the instrument would be applied. The final instrument consisted of the following eight questions: (1) Compared with other people your age, the appearance of your gums is? (2) In the past year, have you had bleeding gums when brushing your teeth? (3) In the past year, have you had injured or infected gums? (4) In the past year, have you had bad breath? (5) In the past year, have you had tooth pain? (6) Have you lost any tooth? (7) Have you noticed any teeth or molars moving? (8) Have you ever had a tooth or molar come loose and then fall out? All responses had a dichotomous scale (0=no/1=yes), except for the first question, which had a Likert‐type response scale (excellent/very good/good/fair/bad). For this question, the responses were recategorized dichotomously (0=excellent/very good/good and 1=fair/bad).

### 2.4 Clinical Variables of Oral Health Status

The measurement of plaque, dental calculus, and pocket depth at probing was performed by two previously standardized dentists (kappa coefficient ≥ 0.90). Artificial light and dental mirror #5 were used. Dental bacterial plaque and dental calculus were assessed at the mesial, distal, vestibular, and lingual sites of all teeth present according to the Green and Vermillion criteria. The percentage of plaque‐covered sites was calculated; hygiene was classified as “good” or “poor,” respectively, according to whether < 10% or ≥ 10% of plaque‐covered surface sites were found on at least 2/3 of the tooth surface. Similarly, for dental calculus, hygiene was classified as “good” or “poor,” respectively, according to whether < 10% or ≥ 10% of calculus‐covered surface sites were found on at least 1/3 of the tooth surface. The Oral Hygiene Index (OHI) was constructed by adding the number of sites with surfaces covered with > 2/3 plaque or > 1/3 dental calculus divided by the total number of surfaces present. Hygiene was classified as “good” with < 10% and “poor” with ≥ 10% of surfaces covered. Periodontal status was assessed with a WHO periodontal probe (Hu‐Friedy®), using the Periodontal Screening and Recording Index (PSR). MP was defined as having at least one tooth with a score of 3 (3.5–5.5 mm depth at probing), and SP with a score of 4 (> 5.5 mm depth at probing) and furcation involvement or gingival recession ≥ 3.5 mm (Dye and Selwitz 2005).

### 2.5 Statistical Analysis

All analyses were performed based on the severity of periodontitis. Initially, an analysis of sociodemographic variables, self‐reported periodontal status, and clinical variables was performed using Pearson's chi‐square test for categorical variables and Wilcoxon's rank sum test for continuous variables.

To determine construct validity, an exploratory factor analysis was performed. Sample adequacy was evaluated with the Kaiser–Meyer–Olkin (KMO) test (> 0.5) (Kaiser 1974), the correlation matrix test (> 0.01), and the Bartlett sphericity test (*p* ≤ 0.05) (Bartlett 1954). The principal component method was used for the extraction of the factors, keeping the factors with eigenvalues ≥ 1 according to the KMO criterion. To simplify the interpretation of the factors, the Varimax orthogonal rotation method was used. Each item was assigned to the factor in which it presented its highest load. Items exceeding in more than one factor the established value ≥ 0.40 or with factor loads < 0.40 would be removed from the instrument in search of a simple structure (Fabrigar and Wegener 2011). Internal consistency for each of the factors was assessed with Cronbach's alpha.

The predictive validity for each factor and the complete instrument was assessed using logistic regression models, with MP and SP clinically measured as the gold standard. Statistics for the AUC‐ROC and 95% confidence interval (95% CI) were obtained. AUC‐ROC values < 0.7 were considered low, 0.7–0.9 moderate, and > 0.9 high (Metz 1978). In addition, sensitivity and specificity values were determined and defined as low (< 60%), moderate (60%–79%), and high (> 80%) (Yore et al. 2007). The cutoff point of the predicted probability to identify periodontitis was established according to the observed prevalence of MP (0.45) and SP (0.25) in the sample studied. Positive (LR +) and negative (LR‐) likelihood ratios were also calculated. Models for periodontitis were adjusted for age, smoking, schooling, and diabetes. Sex and area of residence did not show differences in the model, so they were not included. All analyses were performed using Stata/SE 14 (Stata Corp, College Station, TX, USA).

## 3 Results

### 3.1 Description of the Study Population

The percentage of MP and SP clinically measured was 49.3% and 28.9%, respectively. People with MP and SP were older and had a higher percentage of residence in rural areas, self‐report of indigenous origin, and no schooling compared with those without periodontitis. (Table 1) Participants with MP and SP had a significantly lower percentage of dental visits in the past 12 months, tooth brushing two or more times a day, greater self‐perception of bleeding gums when tooth brushing, swollen or infected gums, tooth mobility, tooth loss after loosening, and poor oral hygiene compared with those without periodontitis (Table 2).

**Table 1:** Sociodemographic and clinical characteristics according to periodontal clinical condition of adults from Comitán de Domínguez, Chiapas 2013.

	No periodontitis *n* = 99 (%)	Moderate periodontitis *n* = 224 (%)	Severe periodontitis *n* = 131 (%)	*p* * value
Age, median (p25–p75)	35 (27–47)	39 (31–49)	48 (37–58)	< 0.001
Sex				
Women	79 (79.8)	155 (69.2)	95 (72.5)	0.145
Men	20 (20.2)	69 (30.8)	36 (27.5)	
Area of residence				
Urban	33 (33.3)	49 (21.9)	25 (19.1)	0.029
Rural	66 (66.7)	175 (78.1)	106 (80.9)	
Indigenous origin				
No	43 (43.4)	100 (44.6)	53 (40.5)	< 0.001
Yes	56 (56.6)	124 (55.4)	78 (59.5)	
Schooling				
≥ Primary	72 (72.7)	130 (58.0)	62 (47.3)	0.001
No	27 (27.3)	94 (42.0)	69 (52.7)	
Occupation				
Housekeeper	71 (71.7)	136 (60.7)	85 (64.9)	0.073
Farmer	13 (13.1)	57 (25.5)	34 (25.9)	
Merchant‐Other	15 (7.2)	31 (13.8)	12 (9.2)	
Diabetes				
No	90 (90.9)	212 (94.6)	127 (96.9)	0.137
Yes	9 (9.1)	12 (5.4)	4 (3.1)	
Tobacco use				
Has never smoked	78 (78.8)	178 (79.5)	101 (77.1)	0.461
Ex‐smoker	13 (13.1)	18 (8.0)	12 (9.2)	
Current smoker	8 (8.1)	28 (12.5)	18 (13.7)	

*Note:* Number and percentage were used unless otherwise indicated.

The Wilcoxon rank sum test was used for the comparison of medians.

* Proportions were compared using Pearson's chi‐square test.

**Table 2:** Oral health status according to periodontal clinical condition of adults from Comitán de Domínguez, Chiapas 2013.

Variables	No periodontitis *n* = 99 (%)	Moderate periodontitis *n *= 224 (%)	Severe periodontitis *n* = 131 (%)	*p* * value
Dental visit in the previous 12 months				
Yes	6 (6.1)	11 (4.9)	7 (5.4)	0.182
No	93 (93.9)	213 (95.1)	124 (96.6)	
Frequency of tooth brushing per day				
Two or more times	72 (72.7)	127 (56.7)	73 (55.7)	0.013
One time or less	27 (27.3)	97 (43.3)	58 (44.3)	
*Self‐reported periodontal status*				
Compared with other people your age, the appearance of your gums is?				
Good‐excellent	68 (69.4)	151 (67.4)	84 (64.1)	0.685
Bad‐fair	30 (30.6)	73 (32.6)	47 (35.9)	
In the past year, have you had bleeding gums when brushing your teeth?				
No	61 (62.2)	120 (53.6)	58 (44.3)	0.025
Yes	37 (37.8)	104 (46.4)	73 (55.7)	
In the past year, have you had injured or infected gums?				
No	70 (71.4)	174 (77.7)	83 (63.4)	0.014
Yes	28 (28.6)	50 (22.3)	48 (36.4)	
In the past year, have you had bad breath?				
No	52 (53.0)	114 (50.9)	62 (47.3)	0.673
Yes	46 (47.0)	110 (49.1)	69 (52.7)	
In the past year, have you had tooth pain?				
No	57 (57.6)	127 (56.7)	69 (52.7)	0.698
Yes	42 (42.4)	97 (43.3)	62(47.3)	
Have you lost any tooth?				
No	45 (45.9)	94 (42.0)	46 (35.1)	0.230
Yes	53 (54.1)	130 (58.0)	85 (64.9)	
Have you noticed any teeth or molars moving?				
No	85 (86.7)	185 (82.6)	86 (65.7)	< 0.001
Yes	13 (13.3)	39 (17.4)	45 (34.3)	
Have you ever had a tooth or molar come loose and then fall out?				
No	77 (78.6)	173 (77.2)	80 (61.01)	0.002
Yes	21 (21.5)	51 (22.8)	51 (38.9)	
*Clinical evaluation*				
Number of teeth present (median, p25–p75)	26 (21‐28)	26 (26‐28)	24 (17‐28)	0.012
Dentobacterial plaque				
Proper hygiene	62 (62.6)	73 (32.6)	26 (19.9)	< 0.001
Poor hygiene	37 (37.4)	151 (67.4)	105 (80.1)	
Dental calculus				
Proper hygiene	19 (19.2)	39 (17.4)	23 (17.6)	0.924
Poor hygiene	80 (80.8)	185 (82.6)	108 (82.4)	
Oral Hygiene Index				
Proper hygiene	25 (25.3)	23 (10.3)	13 (9.9)	< 0.001
Poor hygiene	74 (74.7)	201 (89.7)	118 (90.8)	

*Note:* Number and percentage were used unless otherwise indicated.

The Wilcoxon rank sum test was used for the comparison of medians.

* Proportions were compared using Pearson's chi‐square test.

### 3.2 Construct Validity

Factor analysis values showed two factors for MP (eigenvalue = 1.24) and two factors for SP (eigenvalue = 1.33) with a total explained variance of 44.1% and 47.5%, respectively. In both cases, the KMO statistic (MP = 0.6790, SP= 0.6600), the determination of the correlation matrix (MP = 0.421, SP = 0.287), and the Bartlett sphericity test (*p* < 0.001) had values above the minimum standard to perform the factor analysis. When the orthogonal rotation was performed by the Varimax method, all factors presented factorial loads > 0.40, so no item was excluded from the instrument. Regarding the adequacy of the items, factor one was loaded with the first 5 items for MP and the first 4 items for SP (Table 3).

**Table 3:** Factor loads for self‐reported severe periodontitis among adults from Comitán de Domínguez, Chiapas 2013.

	Moderate periodontitis *N* = 224			Severe periodontitis *N* = 131		
Variables	Factor 1	Factor 2	Commonalities	Factor1	Factor 2	Commonalities
1. Compared with other people your age, the appearance of your gums is?	**0.4879**	0.1300	0.7450	**0.4427**	0.1135	0.7911
2. In the past year, have you had bleeding gums when brushing your teeth?	**0.6626**	−0.1152	0.5476	**0.7697**	−0.1667	0.3797
3. In the past year, have you had injured or infected gums?	**0.7559**	0.1297	0.4117	**0.7469**	0.2061	0.3997
4. In the past year, have you had bad breath?	**0.5925**	0.1272	0.6328	**0.6742**	0.1701	0.5165
5. In the past year, have you had tooth pain?	**0.4425**	0.3715	0.6661	0.4854	**0.5280**	0.4857
6. Have you lost any tooth?	0.0081	**0.7253**	0.4739	0.0758	**0.7570**	0.4212
7. Have you noticed any teeth or molars moving?	0.2543	**0.5758**	0.6038	0.1581	**0.5294**	0.6947
8. Have you ever had a tooth or molar come loose and then fall out?	−0.0342	**0.7783**	0.3931	−0.0693	**0.6966**	0.5099

*Note:* The highlighted items indicate the factor with the highest load for each item.

### 3.3 Instrument Reliability

Evaluation of internal consistency for MP at factor 1 and factor 2 resulted in Cronbach's alpha of 0.571 and 0.534, respectively, and for SP of 0.624 and 0.566, respectively.

### 3.4 Predictive Validity

In logistic regression models 1 (items 1–5 + risk factors) and 3 (items 1–8 + risk factors), participants who reported bleeding gums when tooth brushing (OR = 1.92, 95% CI 1.08–3.41) were more likely to have MP than those without bleeding gums. Conversely, participants who reported injured or infected gums were less likely to have MP (OR = 0.45, 95% CI 0.22–0.91) than those without any injury or infection. In the case of SP, in all three models, participants who had bleeding gums when tooth brushing (OR = 3.56, 95% CI 1.60–7.92) and tooth mobility (OR = 3.08, 95% CI 1.5–7.05) were more likely to have SP than those without these conditions.

**Figure 1:**
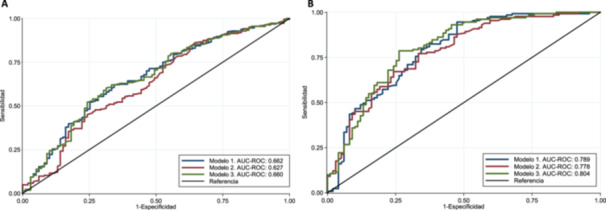
AUC‐ROC of models for the prediction of periodontitis among adult population from the Municipality of Comitán de Domínguez, Chiapas 2013. (A) AUC‐ROC of the three models that predict moderate periodontitis. Model 1 comprises items 1–5 + risk variables; model comprises items 6–8 + risk variables; model 3 comprises items 1–8 + risk variables. (B) AUC‐ROC of the three models that predict severe periodontitis. Model 1 comprises items 1–4 + risk variables; model 2 comprises items 5–8 + risk variables; model 3 comprises items 1–8 + risk variables.

Finally, when predictive validity was evaluated, the models for MP and SP showed high sensitivity values (92.2%–100%) and low specificity values (0%–28.3%). However, the AUC‐ROC for MP was low (model 1, 0.662 [95% CI 0.59–0.72]) compared with the AUC‐ROC for SP, which was moderate (model 3, 0.804 [95% CI 0.746–0.863]) (Table 4 and Figure 1).

**Table 4:** Multiple logistic regression models for the prediction of severe periodontitis among adults from Comitán de Domínguez, Chiapas 2013.

	Moderate periodontitis (*n* = 323; cases = 224)						Severe periodontitis (*n* = 230; cases = 131)					
	Model 1 (Factor 1 + risk variables)		Model 2 (Factor 2 + risk variables)		Model 3 (Full Instrument + risk variables)		Model 1 (Factor 1 + risk variables)		Model 2 (Factor 2 + Risk variables)		Model 3 (Full Instrument + Risk variables)	
	OR (95% CI)	*p* value	OR (95% CI)	*p* value	OR (95% CI)	*p* value	OR (95% CI)	*p* value	OR (95% CI)	*p* value	OR (95% CI)	*p* value
1. Compared with other people your age, the appearance of your gums is?	1.15 (0.66–2.02)	0.611	—	—	1.11 (0.63–1.98)	0.699	0.69 (0.33–1.41)	0.310	—	—	0.63 (0.30–1.36)	0.247
2. In the past year, have you had bleeding gums when brushing your teeth?	1.93 (1.08–3.42)	0.025	—	—	1.92 (1.08–3.41)	0.026	3.46 (1.60–7.46)	0.001	—	—	3.56 (1.60–7.92)	0.002
3. In the past year, have you had injured or infected gums?	0.45 (0.22–0.91)	0.025	—	—	0.45 (0.22–0.91)	0.027	0.85 (0.38–1.89)	0.697	—	—	0.96 (0.40–2.26)	0.929
4. In the past year, have you had bad breath?	1.15 (0.66–2.03)	0.606	—	—	1.15 (0.65–2.02)	0.618	0.64 (0.32–1.28)	0.214	—	—	0.76 (0.36–1.58)	0.468
5. In the past year, have you had tooth pain?	0.85 (0.49–1.48)	0.580			0.84 (0.47–1.49)	0.554	—	—	0.64 (0.32–1.27)	0.212	0.52 (0.24–1.14)	0.105
6. Have you lost any tooth?	—	—	1.01 (0.57–1.76)	0.968	1.05 (0.58–1.89)	0.864	—	—	0.61 (0.28–1.32)	0.217	0.62 (0.28–1.39)	0.254
7. Have you noticed any teeth or molars moving?	—	—	1.23 (0.60–2.54)	0.561	1.24 (0.58–2.62)	0.570	—	—	2.75 (1.23–6.16)	0.013	3.08 (1.35–7.05)	0.008
8. Have you ever had a tooth or molar come loose and then fall out?	—	—	0.85 (0.45–1.61)	0.625	0.89 (0.46–1.72)	0.746	—	—	1.57 (0.76–3.22)	0.217	1.24 (0.57–2.66)	0.577
Precision Measurements*												
% Sensitivity	97.3		100		97.3		99.2		98.5		96.9	
% Specificity	2.0		0		3.0		28.3		15.5		28.3	
RV+	0.99		1.00		1.00		1.38		1.17		1.35	
RV–	1.35		0		0.9		0.03		0.09		0.10	
AUC‐ROC (95% CI)	0.662 (0.59–0.72)		0.627 (0.55–0.69)		0.660 (0.595–0.725)		0.789 (0.729–0.850)		0.778 (0.718–0.839)		0.804 (0.746–0.863)	

*Note:* All models were adjusted for age, smoking, schooling, and diabetes. Sensitivity and specificity values for moderate and severe periodontitis are based on the predicted probability of being a case at a cut‐off point of 0.45 and 0.25, respectively. This point was considered by rounding the prevalence observed by the examiners for both conditions to the nearest lower value.

Abbreviations: AUC‐ROC, area under the receiver operating characteristic curve; CI, confidence interval; OR, odds ratio; RV–, negative likelihood ratio; RV+, positive likelihood ratio.

## 4 Discussion

In the present study, we found that the items of the instrument correctly represent two factors for MP (factor 1, items 1–5; factor 2, items 6–8) and SP (factor 1, items 1–4; factor 2, items 5–8) about the probability of suffering periodontitis. Although the models generally showed high sensitivity values, the AUC‐ROC performed better for SP prediction. The use of this instrument can be an alternative to identify people with the disease, simplify the collection of information, and improve periodontitis surveillance.

As far as we know, only Wright et al. (2021) have performed an analysis of the internal structure of a set of 22 questions on periodontal health. Their results were similar to ours as to the grouping of the items into two factors, which they named “physiological” and “functional” according to the severity of the disease. Similarly, in our study, the first factor comprised items related to early stages of the disease (change in gum color and texture, bleeding, halitosis), whereas the second identified signs and symptoms of more severe stages of periodontitis (tooth mobility, loss of a tooth with previous mobility), where functional issues are affected and the symptoms are more noticeable (Wright et al. 2021). On the other hand, in this study, only the item “In the last year, have you had pain in your teeth?” went from factor 1 in MP to factor 2 in SP. However, it has been observed that in the initial stages of periodontitis pain is more related to the inflammatory process of the gums, and in severe phases to tooth mobility caused by the destruction of periodontal support (Costa et al. 2023).

In addition, factor analysis allowed us to identify a group of questions that can account for the severity of periodontitis. Other authors have only assessed the sensitivity and specificity of individual items (Eke and Dye 2009; Abbood et al. 2016); yet, using a single self‐report measure can confuse discrimination. For example, gingival bleeding may be reduced in people who smoke due to the vasoconstriction generated by nicotine; hence, the presence of periodontitis would be underestimated considering only this variable as a predictor of the disease (Spiekerman et al. 2003). Such limitation was demonstrated in a study conducted in Thailand, where a high rate of false negatives was observed in patients who had gum inflammation and self‐reported having periodontitis (AUC‐ROC = 0.56) (Lertpimonchai et al. 2023). Using factor analysis to group items and represent common factors was therefore an appropriate alternative to have a valid instrument.

The internal consistency of the instrument was assessed using Cronbach's alpha. Although it is agreed that an adequate value should be ≥ 0.70 (Jisu et al. 2006; Nunnally and Bernstein 1994), some authors have shown that values ≥ 0.50 are acceptable in exploratory phases of the design process, as long as the items measure the same construct and each one contains ≥ 3 items (Schmitt 1996; Streiner et al. 2015). In this study, Cronbach's alpha was calculated after factor analysis, thus ensuring that the items evaluated the same content and showed adequate homogeneity in each factor.

Finally, the models for SP had better predictive values than those for MP. The few or no noticeable symptoms in MP patients may explain the low ability to identify people in earlier stages of periodontitis. Yet, as the disease evolves, more objective indicators such as mobility, tooth loss, and dental pain appear. Symptoms increase as well with age and factors such as smoking and chronic comorbidities (Buset et al. 2016). For instance, in the present study, bleeding during tooth brushing, tooth mobility, and all risk variables were associated with SP. These results coincide with those of different studies conducted in the United States of America, Spain, and Brazil (Eke and Dye 2009; Montero et al. 2020; Reiniger et al. 2020), where questions about gingival bleeding and tooth mobility were more sensitive and specific to predict SP (sensitivity 0.89; specificity 0.82, AUC‐ROC = 0.86) than MP (sensitivity 0.77; specificity 0.67, AUC‐ROC = 0.72; Reiniger et al. 2020).

An advantage of this study is that the PSR assessment was performed on all teeth pre,sent, which lessens the likelihood of underestimating the clinically determined prevalence of periodontitis. In addition, during the training, the intra‐ and inter‐examiner correlation in the clinical evaluation was verified to strengthen validation. Another advantage was the use of a cut‐off point for the predicted probability to identify a case of periodontitis, so the likelihood of misclassification was reduced. A limitation is that questions related to periodontal treatment were not asked; also, neither a diagnosis made by a dentist, nor the use of cleaning aids (dental floss, interproximal brushes, and mouthwash) was included, since the largest proportion of the population in this study had very low access to oral health services and did not know about dental cleaning tools.

In conclusion, self‐reported measures of periodontitis represented the initial and outcome stages of the disease in two factors, with greater relevance to predict the risk of SP. The instrument used may be an accessible and low‐cost alternative for surveillance of periodontitis at population level by nondental personnel. The ultimate goal is to identify the population at risk in settings where access to oral health services is limited, which causes a negative impact on the early diagnosis and timely treatment of periodontitis.

## Author Contributions

José A. Falcón‐Flores contributed to the data analysis, wrote, and reviewed the manuscript, and contributed to the discussion. María E. Jiménez‐Corona contributed to the design of the study, reviewed the manuscript, and contributed to the discussion. Ileana G Rangel‐Nieto collected the data, reviewed the manuscript, and contributed to the discussion. Marisela Vazquez‐Duran reviewed the manuscript and contributed to the discussion. Aida Jiménez‐Corona designed the study, contributed to the data analysis, reviewed the manuscript, and contributed to the discussion. In addition, she is responsible for this investigation and takes responsibility for the integrity of the data and accuracy of the data analysis.

## Ethics Statement

The Research, Ethics and Biosafety Committees of the National Institute of Public Health approved the study protocol. All participants signed an informed consent form.

## Conflicts of Interest

The authors declare no conflicts of interest.

## Data Availability

The authors have nothing to report.
